# Effect of effervescent multivitamin solutions on the color stability of contemporary resin composites: an in vitro study

**DOI:** 10.2340/biid.v13.46591

**Published:** 2026-08-04

**Authors:** Yeliz Hayriye Yazıcıoğlu Pirpir, Osman Tolga Harorlı

**Affiliations:** Department of Restorative Dentistry Faculty of Dentistry, Akdeniz University, Antalya, Turkey

**Keywords:** Color, coloring agents, resin composites, dental esthetics, vitamins

## Abstract

**Objective:**

Given the limited evidence on the effect of effervescent multivitamin solutions on the color stability of contemporary resin composites, this study aimed to evaluate and compare their impact under simulated daily use conditions.

**Materials and Methods:**

Five resin composites (Charisma Smart, Filtek One Bulk Fill, Omnichroma, Ruby Comp Nano, Ruby Flow) were tested using 200 disk-shaped specimens divided into a distilled water control group and three experimental groups: Sambucol Plus (SMB), Supradyn Effervescent Tablet (SPR), and Youplus Vitamin C, Zinc, Propolis (YPL) effervescent tablets. Each tablet was dissolved in 200 mL distilled water; specimens were immersed twice daily for 2 min over 28 days. Color measurements (CIELAB, ΔE_00_, Whiteness Index) were taken at baseline, 14 and 28 days using a spectrophotometer. Data were analyzed using repeated-measures analysis of variance (ANOVA); Welch ANOVA and Games–Howell post-hoc tests were used where appropriate, with the significance level set at *p* < 0.05.

**Results:**

Resin composite and immersion solution had significant effects on color change (*p* < 0.001), while time interval was also significant (*p* = 0.009). After 28 days, the highest discoloration was observed in Filtek One Bulk Fill immersed in Youplus (ΔE_00_ = 9.93 ± 0.73), followed by Omnichroma immersed in Supradyn (ΔE_00_ = 5.63 ± 0.83). Several groups exceeded the clinical acceptability threshold (ΔE_00_ = 1.8), particularly Filtek One Bulk Fill and Omnichroma exposed to Youplus and Supradyn. Significant time × composite, time × solution, and time × composite × solution interactions were detected (all *p* < 0.001). The Whiteness Index also showed significant changes over time, particularly in the Youplus- and Supradyn-exposed groups.

**Conclusion:**

Within the limitations of this in vitro study, multivitamin effervescent tablets adversely affected the color stability of some resin composites, particularly Filtek One Bulk Fill and Omnichroma. Further clinically relevant and in vivo studies are needed to determine the significance of these findings under oral conditions.

## Introduction

Changes in the color of dental restorations, particularly those in the anterior region, are a common concern among patients, prompting consultations with dental professionals [1]. The color stability of resin composites is influenced by a variety of factors including water absorption, surface roughness, and the processes of finishing and polishing [2–4]. Other significant factors are the methods and degree of polymerization, surface hardness, and the patient’s oral hygiene and dietary habits, which collectively determine the duration of the material’s exposure in the oral environment [5, 6].

However, staining remains a substantial esthetic challenge. It typically manifests as surface staining, edge staining from microleakage, changes in surface morphology due to wear, and degradation of the material’s content [7, 8]. Despite routine oral hygiene practices aimed at reducing surface staining, intrinsic discoloration is largely determined by the composition of the resin composites and is challenging to manage [9].

Vitamin supplements, available in various forms including syrups, pills, chewable, lozenges, and effervescent tablets, often contain acidic components that can influence dental materials [10, 11]. Effervescent vitamins combine acids such as citric or tartaric acid with alkali metal carbonate salts and various vitamins and minerals. This combination, while enhancing the solubility and effectiveness of the ingredients, poses potential risks for dental erosion and color alteration in resin composites [10]. Specifically, organic acids such as citric acid are known to soften the polymer matrix of resin composites, potentially leading to the dislodgment of filler particles and an increase in surface roughness [12, 13]. An augmented surface roughness, in turn, provides a larger area for pigment accumulation, thereby accelerating extrinsic discoloration [14].

While the erosive effects of various acidic beverages on dental composites have been extensively documented [15, 16], there is a notable gap in the literature regarding the specific impact of increasingly popular multivitamin effervescent tablets on color stability. The current lack of specific research makes it difficult for clinicians to offer evidence-based guidance on the possible esthetic impact of these supplements.

The purpose of this study is to evaluate the effects of multivitamin effervescent tablets on the color stability of various dental resin composites. These findings may assist dental professionals in better understanding and anticipating color stability outcomes in composite restorations, thereby enhancing their ability to meet patient expectations and extend the longevity of esthetic restorations. The null hypothesis of this study is that different multivitamin effervescent tablets have no effect on the color change of resin composites.

## Materials and methods

### Composite materials

**Figure 1:**
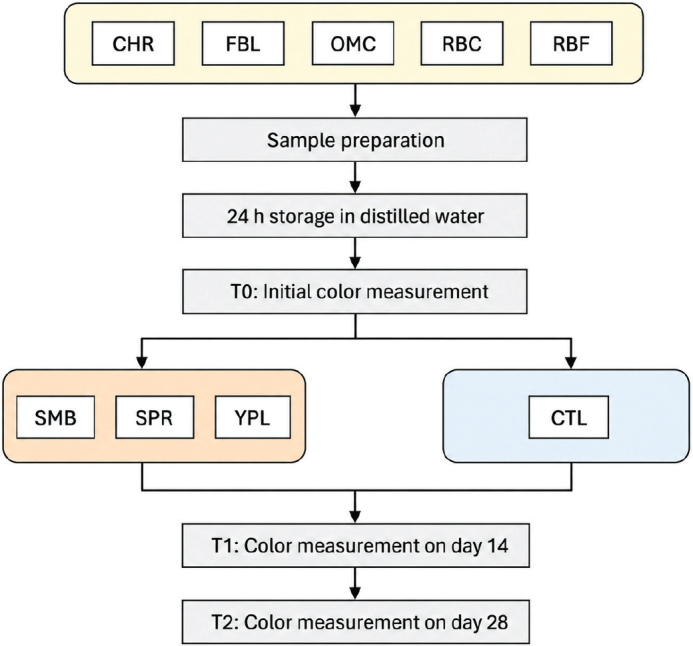
Flowchart illustrating the grouping, immersion protocol, and color measurement timeline used to assess the effects of multivitamin solutions on the color stability of five resin composites. The tested resin composites were Charisma Smart (CHR), Filtek One Bulk Fill (FBL), Omnichroma (OMC), Ruby Comp Nano (RBC), and Ruby Flow (RBF). The immersion solutions were distilled water control (CTL), Sambucol Plus (SMB), Supradyn Effervescent Tablet (SPR), and Youplus Vitamin C Zinc Propolis effervescent tablet (YPL).

This study utilized five resin composites and three multivitamin effervescent tablets. The study flowchart is shown in Figure 1. The resin composites used in the study, including their types, matrix content, filler particle sizes, filler loadings, colors, and lot numbers as defined by their manufacturers, are detailed in Table 1. Except for Omnichroma, which is a single-shade resin composite, the A2 shade was selected because it represents a commonly used clinical shade and allowed standardized comparison across the tested materials.

**Table 1:** Details of the resin composite materials used in the study.

Composite/shade	Abbreviation	Type	Resin matrix	Filler characteristics	Filler loading	Manufacturer	Lot number
Charisma Smart/A2	CHR	Submicrohybrid resin composite	TEGDMA, oxybenzone, methacrylic acid, 2,6-di-tert-butyl-p-cresol	Barium aluminum fluoride glass and silicon dioxide; particle size: 0.005–10 µm	59 vol%	Heraeus Kulzer GmbH, Hanau, Germany	M010538
Filtek One Bulk Fill/A2	FBL	Bulk-fill resin composite	AFM, AUDMA, UDMA, and 1,12-dodecane-DMA	Silica and zirconia nanomers (4–20 nm), zirconia/silica nanoclusters (0.6–1.4 µm), and ytterbium trifluoride particles (~100 nm)	76.5 wt%; 58.5 vol%	3M ESPE, St. Paul, MN, USA	NE55682
Omnichroma/One shade	OMC	Supra-nanofill resin composite	UDMA and TEGDMA	Supra-nano spherical SiO_2_–ZrO_2_ fillers; particle size: 260 nm	79 wt%; 68 vol%	Tokuyama Dental, Tokyo, Japan	076E12
Ruby Comp Nano/A2	RBC	Nanohybrid resin composite	Bis-GMA and methacrylate polymers	Inorganic fillers; particle size: 0.02–0.7 µm	80 wt%	Incidental, Istanbul, Turkey	RCYA2152
Ruby Flow/A2	RBF	Flowable resin composite	Methacrylate polymers	Inorganic fillers; particle size: 0.02–0.7 µm	60 wt%	Incidental, Istanbul, Turkey	RF1270

CHR: Charisma Smart; FBL: Filtek One Bulk Fill; OMC: Omnichroma; RBC: Ruby Comp Nano; RBF: Ruby Flow; WID: Whiteness Index for Dentistry; CTL: distilled water control; SMB: Sambucol Plus; SPR: Supradyn Effervescent Tablet; YPL: Youplus Vitamin C, Zinc, Propolis effervescent tablet.

### Multivitamin effervescent tablets

Three commercially available effervescent multivitamin products were selected for this study: Sambucol Plus effervescent tablet (SMB), Supradyn Effervescent Tablet (SPR), and Youplus Vitamin C Zinc Propolis effervescent tablet (YPL). Detailed information regarding the selected products is presented in Table 2.

**Table 2:** Details of multivitamin effervescent tablets.

Product/abbreviation	Composition	Manufacturer	Lot number	Initial pH
Sambucol Plus Effervescent Tablet (SMB)	Acidity regulator: citric acid; bulking agent: sodium hydrogen carbonate; maltodextrin; coloring agent: beetroot red; L-ascorbic acid (vitamin C); flavoring agents: strawberry and raspberry flavors; sweetener: sucralose; zinc sulfate; stabilizers: sorbitol and polyvinylpyrrolidone; black elderberry fruit extract	PharmaCare Europe, West Sussex, UK	2010106	4.4
Supradyn Effervescent Tablet (SPR)	Acidity regulator: sodium hydrogen carbonate; acid: citric acid; bulking agents: sorbitol, calcium carbonate, magnesium sulfate, isomalt, and magnesium carbonate; L-ascorbic acid; coloring agents: carotenes and beetroot red; sodium carbonates; ferric pyrophosphate; coenzyme Q10; flavoring agents: orange and passion fruit flavors; zinc citrate; sweetener: aspartame; sodium selenite; D-pantothenate calcium; manganese sulfate; cholecalciferol	Bayer, Basel, Switzerland	N3A927	4.5
Youplus Vitamin C Zinc Propolis Effervescent Tablet (YPL)	L-ascorbic acid, 1,000 mg vitamin C; acid regulators: sodium carbonates, citric acid, and malic acid; sweeteners: isomalt, aspartame, and acesulfame K; coloring agent: carotenes; carrier: polyethylene glycol; zinc citrate; pure propolis; lemon flavor	Abdi Ibrahim, Istanbul, Turkey	22L279	3.9

Initial pH values were measured immediately after each effervescent tablet was dissolved in 200 mL of distilled water.

### Sample size calculation

An *a priori* sample size calculation was performed using G*Power software version 3.1.9.7. The calculation was based on repeated-measures analysis of variance (ANOVA) with within–between interaction, considering two repeated ΔE_00_ measurements (T0–T1 and T0–T2), 20 experimental groups, an alpha level of 0.05, a statistical power of 0.80, a correlation among repeated measures of 0.50, and a non-sphericity correction of 1. A medium effect size was assumed (*f* = 0.25) based on previous in vitro color stability studies and clinically relevant CIEDE2000 color differences. The minimum required total sample size was calculated as 100 specimens. Therefore, the use of 200 specimens, with 10 specimens in each subgroup, was considered sufficient for the multifactorial study design.

### Composite specimen preparation

A total of 40 disks (5 mm diameter, 2 mm depth) were prepared for each of five different resin composites (*n* = 200). During specimen preparation, a custom Teflon mold was employed, with Mylar strips (Mylar Type D, DuPont, DE, USA) placed on both its upper and lower surfaces. The Mylar strips provided smooth, standardized specimen surfaces and uniform thickness while minimizing variability associated with finishing and polishing procedures. The designated resin composite was then introduced into the mold cavity. A second translucent polyester strip was positioned on top of the Teflon mold to create a confined space for the resin composite. Light-curing polymerization was achieved by exposing the top surface of the resin composite to a LED light-curing unit (Valo Ultradent Products, UT, USA) for a standardized duration of 20 s. The light source delivered an irradiance of 1,000 mW/cm^2^ and was maintained in direct contact with the Mylar strip to ensure consistent light-curing distance across all specimens. To ensure consistent irradiance output throughout the study, the light-curing unit was calibrated using a dental radiometer (Kerr Demi Ultra radiometer, Kerr Corporation, Orange, California, USA) before commencing the study and verified after every five polymerization cycles.

Following the light-curing process, the prepared specimens were submerged in distilled water for a period of 24 h. This standardized storage period facilitated initial color measurements before further experimentation.

Color measurements were performed using the CIELAB color space. The L (lightness), a (green – red), and b (blue – yellow) values were recorded for each specimen at the baseline (T0). A clinical spectrophotometer (VITA Easyshade V, VITA Zahnfabrik, Bad Säckingen, Germany) was used for color measurements.

Following baseline color measurements, specimens within each resin composite group were randomly allocated to the four immersion solutions (*n* = 10 per subgroup) using a computer-generated randomization sequence. Specimens were labeled with numerical codes, and group identities were concealed from the operator performing color measurements. One subgroup from each resin type served as a control, exposed to distilled water. The operator performing the color measurements was blinded to group allocation; and all measurements were obtained using a standardized spectrophotometric protocol.

### Preparation of solutions and storage procedure

To simulate real-life multivitamin consumption, each solution was prepared by dissolving one effervescent tablet in 200 mL of water, following the respective manufacturer’s instructions. This immersion was performed twice daily for 2 min each time, over a total period of 28 days. After each immersion cycle, the specimens were rinsed with distilled water and stored in distilled water until the next cycle. Solutions were freshly prepared before each immersion. Specimens in the control group were kept in distilled water which was renewed daily. Throughout the study period, all specimens were stored in distilled water at 37°C, except during the immersion procedures in the test solutions.

### Color measurements and calculations

As stated above, color measurements were performed using a clinical spectrophotometer, which is widely recognized for its clinical accuracy. Nevertheless, factors such as specimen surface geometry, translucency, and operator positioning may affect measurement precision [17]. To mitigate these influences and ensure data integrity, a rigorous standardized measurement protocol was implemented. This protocol included precise specimen preparation, perpendicular placement of the probe, and averaging of three consecutive readings per specimen to enhance measurement reliability. All composite disks were measured on a matte black background to provide a consistent, high-contrast base. This non-reflective surface minimizes light scatter, ensuring the recorded color originates from the specimen itself. All measurements were performed from the central area of the upper surfaces of the disks. The measurements were based on the Commission Internationale de l’Eclairage (CIE) Lab* system. The spectrophotometer was standardized according to the manufacturer’s instructions. Three repeated readings were taken for each specimen, and the average L*, a*, and b* values were obtained. Color measurements were repeated on the 14th (T1) and 28th days (T2) of the study in the same manner, and the obtained values were recorded. Measurements were taken after the specimens were removed from the solutions, rinsed with distilled water, and gently dried. Color change values were then calculated using the ΔE_00_ formula.

ΔE_00_ = √[(ΔL'/KLSL)² + (ΔC'ab/KCSC)² + (ΔH'ab/KHSH)² + RT(ΔC'ab/KCSC)(ΔH'ab/KHSH)]

ΔL*, ΔC*, and ΔH* represent lightness, chroma, and hue differences, respectively. RT indicates the interaction between chroma and hue differences. KL, KC, and KH are parametric factors and were set to 1. A perceptibility threshold was established at ΔE_00_ = 0.8, whereas the clinical acceptability threshold was set at ΔE_00_ = 1.8 [18].

The whiteness of the resin composite specimens was quantified using the Whiteness Index for Dentistry (WI_D_) [19], a customized index developed specifically for dentistry based on the CIELAB color space. Color measurements were obtained using a spectrophotometer, and the corresponding CIE L*, a*, and b* values were recorded for each specimen at baseline (T0), after 14 days of immersion (T1), and after 28 days of immersion (T2). The WI_D_ values were calculated according to the formula:

WI_D_ = 0.511L* − 2.324a* − 1.100b*

where L* represents lightness, a* represents the red–green axis, and b* represents the yellow–blue axis. Higher WI_D_ values indicate increased perceived whiteness, whereas lower values indicate darker or more chromatic appearances.

### Data visualization with RGB values

The color changes were visualized by converting the Lab values to Red, Green, Blue (RGB) values using a Python script. The script utilized the matplotlib library to create color swatches representing the measured Lab values [20]. The conversion process involved the following steps:

- Lab to XYZ Conversion: The Lab values were converted to XYZ coordinates using the standard formulas with adjustments for non-linear values.
- XYZ to RGB Conversion: The XYZ values were transformed to RGB values using the following matrix.
- Gamma Correction: The RGB values were adjusted using gamma correction.
- Clipping: Finally, the RGB values were clipped to the range (0, 1).

### pH measurements of solutions

pH measurements of the multivitamin solutions were conducted using a Mettler Toledo T50 pH meter (Mettler Toledo, Schwerzenbach, Switzerland). Measurements were performed only once at the beginning of the experiment, immediately after the solutions were thoroughly mixed to ensure homogeneity and before the specimens were placed. This timing allowed accurate assessment of the initial pH values without interference from the specimens.

The device was calibrated before each session using standard buffer solutions. All measurements were conducted at 25 ± 1°C.

### Statistical analysis

Statistical analyses were performed using jamovi software version 2.6.44 (The jamovi project, Sydney, Australia). Descriptive statistics were calculated as mean, standard deviation (SD), interquartile range (IQR), and 95% confidence interval (CI).

For ΔE_00_ values, repeated-measures ANOVA was performed with time interval as the within-subject factor and resin composite and immersion solution as between-subject factors. The within-subject factor consisted of two ΔE_00_ intervals: T0–T1 and T0–T2. Resin composite had five levels: Charisma Smart, Filtek One Bulk Fill, Omnichroma, Ruby Comp Nano, and Ruby Flow. Immersion solution had four levels: distilled water control, Sambucol Plus, Supradyn Effervescent Tablet, and Youplus Vitamin C Zinc Propolis effervescent tablet.

For WI_D_ analysis, ΔWI_D_ values were calculated by subtracting baseline WI_D_ values from the 14-day and 28-day WI_D_ values. Thus, two ΔWI_D_ intervals were obtained: T0–T1 and T0–T2. The ΔWI_D_ values were compared among resin composite–solution combinations separately for the T0–T1 and T0–T2 intervals.

The normality of residuals was assessed using the Shapiro–Wilk test and Q–Q plots. Homogeneity of variances was evaluated using Levene’s test. Sphericity was assessed using Mauchly’s test for repeated-measures analyses. For the ΔE_00_ analysis, the within-subject factor had only two levels; therefore, the sphericity assumption was automatically satisfied.

Effect sizes were reported as partial eta squared (ηp²) for repeated-measures ANOVA. When significant effects or interactions were detected, post-hoc comparisons were performed. Because Levene’s test indicated heterogeneity of variances for ΔE_00_ intervals, Games–Howell post-hoc tests were used to compare composite–solution combinations separately for the T0–T1 and T0–T2 intervals.

For ΔWI_D_ values, Levene’s test also indicated heterogeneity of variances; therefore, Welch’s one-way ANOVA was used to compare the composite–solution combinations separately for T0–T1 and T0–T2 intervals. Games–Howell post-hoc tests were performed for pairwise comparisons. Statistical significance was set at α = 0.05.

## Results

### ΔE_00_ color changes

Descriptive statistics for ΔE_00_ color change values according to resin composite, immersion solution, and time interval are presented in Table 3.

**Table 3:** Descriptive statistics of ΔE_00_ color change values according to resin composite, immersion solution, and time interval.

Resin composite	Solution	*n*	ΔE_00_ T0–T1 mean ± SD	95% CI	ΔE_00_ T0–T2 mean ± SD	95% CI
CHR	**CTL**	10	1.69 ± 0.75^ghij^	1.15–2.23	1.82 ± 0.78^efg^	1.26–2.38
CHR	**SMB**	10	1.89 ± 0.65^fgh^	1.43–2.35	0.93 ± 0.37^hi^	0.67–1.19
CHR	**SPR**	10	2.51 ± 0.60^defg^	2.08–2.94	2.18 ± 0.25^def^	2.00–2.36
CHR	**YPL**	10	2.35 ± 0.67^defg^	1.87–2.83	2.64 ± 1.01^cde^	1.92–3.36
FBL	**CTL**	10	1.77 ± 0.40^fghi^	1.48–2.06	1.43 ± 0.47^fgh^	1.09–1.77
FBL	**SMB**	10	1.57 ± 0.44^hij^	1.26–1.88	2.30 ± 0.22^def^	2.14–2.46
FBL	**SPR**	10	3.70 ± 0.63^cde^	3.25–4.15	3.76 ± 0.35^c^	3.51–4.01
FBL	**YPL**	10	7.45 ± 0.31^a^	7.23–7.67	9.93 ± 0.73^a^	9.41–10.45
OMC	**CTL**	10	2.67 ± 0.79^cdefg^	2.10–3.24	2.03 ± 0.72^def^	1.51–2.55
OMC	**SMB**	10	3.78 ± 1.14_bcde_	2.96–4.60	2.77 ± 1.12^cd^	1.97–3.57
OMC	**SPR**	10	5.29 ± 0.23^b^	5.13–5.45	5.63 ± 0.83^b^	5.04–6.22
OMC	**YPL**	10	4.01 ± 1.08^bcd^	3.24–4.78	4.48 ± 0.62^bc^	4.04–4.92
RBC	**CTL**	10	0.76 ± 0.37^jk^	0.50–1.02	0.81 ± 0.22^i^	0.65–0.97
RBC	**SMB**	10	0.62 ± 0.31^jk^	0.40–0.84	1.19 ± 0.53^ghi^	0.81–1.57
RBC	**SPR**	10	2.32 ± 0.30^efg^	2.11–2.53	1.30 ± 0.24^fgh^	1.13–1.47
RBC	**YPL**	10	1.73 ± 0.39^fghi^	1.45–2.01	1.68 ± 0.52^efg^	1.31–2.05
RBF	**CTL**	10	0.87 ± 0.31^jk^	0.65–1.09	1.17 ± 0.63^ghi^	0.72–1.62
RBF	**SMB**	10	0.53 ± 0.26^k^	0.34–0.72	1.12 ± 0.34^ghi^	0.88–1.36
RBF	**SPR**	10	2.01 ± 0.45^fg^	1.69–2.33	2.26 ± 0.52^def^	1.89–2.63
RBF	**YPL**	10	0.98 ± 0.22^ijk^	0.82–1.14	1.54 ± 0.34^fg^	1.30–1.78

CHR: Charisma Smart; FBL: Filtek One Bulk Fill; OMC: Omnichroma; RBC: Ruby Comp Nano; RBF: Ruby Flow; WID: Whiteness Index for Dentistry; CTL: distilled water control; SMB: Sambucol Plus; SPR: Supradyn Effervescent Tablet; YPL: Youplus Vitamin C, Zinc, Propolis effervescent tablet.

Values are presented as mean ± standard deviation and 95% confidence interval. Superscript lowercase letters indicate Games–Howell post-hoc groupings within each time interval. Values sharing at least one common letter within the same time interval are not significantly different from each other (*p* > 0.05). T0–T1 and T0–T2 post-hoc groupings were calculated separately.

**Figure 2:**
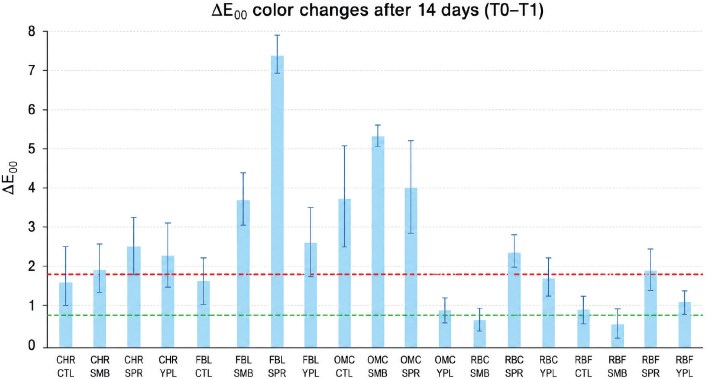
ΔE_00_ Color changes of resin composites after 14 days (T0-T1). The resin composites tested were Charisma Smart (CHR), Filtek One Bulk Fill (FBL), Omnichroma (OMC), Ruby Comp Nano (RBC), and Ruby Flow (RBF). The immersion solutions were distilled water control (CTL), Sambucol Plus (SMB), Supradyn Effervescent Tablet (SPR), and Youplus Vitamin C Zinc Propolis effervescent tablet (YPL). The dashed green line at ΔE_00_ = 0.8 represents the perceptibility threshold, while the red line at ΔE_00_ = 1.8 represents the clinical acceptability threshold.

At the T0–T1 interval (Figure 2), ΔE_00_ values varied markedly among the tested resin composites and immersion solutions. The highest color change was observed in Filtek One Bulk Fill exposed to Youplus Vitamin C Zinc Propolis effervescent tablet (FBL–YPL; 7.45 ± 0.31), followed by Omnichroma exposed to Supradyn Effervescent Tablet (OMC–SPR; 5.29 ± 0.23), Omnichroma exposed to Youplus Vitamin C Zinc Propolis effervescent tablet (OMC–YPL; 4.01 ± 1.08), and Omnichroma exposed to Sambucol Plus (OMC–SMB; 3.78 ± 1.14). In contrast, the lowest ΔE_00_ values were recorded for Ruby Flow and Ruby Comp Nano exposed to Sambucol Plus or distilled water control.

**Figure 3:**
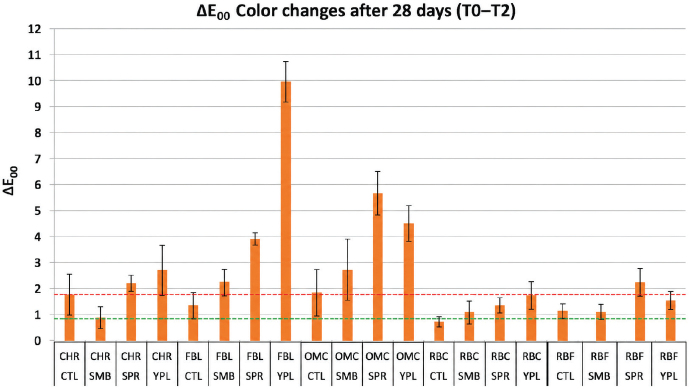
ΔE_00_ Color changes of resin composites after 28 days (T0-T2). The resin composites tested were Charisma Smart (CHR), Filtek One Bulk Fill (FBL), Omnichroma (OMC), Ruby Comp Nano (RBC), and Ruby Flow (RBF). The immersion solutions were distilled water control (CTL), Sambucol Plus (SMB), Supradyn Effervescent Tablet (SPR), and Youplus Vitamin C Zinc Propolis effervescent tablet (YPL). The dashed green line at ΔE_00_ = 0.8 represents the perceptibility threshold, while the red line at ΔE_00_ = 1.8 represents the clinical acceptability threshold.

At the T0–T2 interval (Figure 3), a similar material- and solution-dependent pattern was observed. Filtek One Bulk Fill exposed to Youplus Vitamin C Zinc Propolis effervescent tablet showed the highest ΔE_00_ value (9.93 ± 0.73), followed by Omnichroma exposed to Supradyn Effervescent Tablet (5.63 ± 0.83) and Omnichroma exposed to Youplus Vitamin C Zinc Propolis effervescent tablet (4.48 ± 0.62). Several groups, particularly Filtek One Bulk Fill and Omnichroma exposed to Youplus Vitamin C Zinc Propolis effervescent tablet or Supradyn Effervescent Tablet, exceeded the CIEDE2000 clinical acceptability threshold of ΔE_00_ = 1.8. Ruby Comp Nano and Ruby Flow generally showed lower color changes, especially in the distilled water control and Sambucol Plus groups.

Repeated-measures ANOVA showed that ΔE_00_ values were significantly affected by time interval, resin composite, immersion solution, and their interactions. Significant interaction effects indicated that color change progression differed depending on both the resin composite and the immersion solution. The detailed repeated-measures ANOVA results, including F values, degrees of freedom, *p*-values, and effect sizes, are presented in Table 4.

**Table 4:** Repeated-measures ANOVA results for ΔE_00_ values.

Effect	Sum of squares	*df*	Mean square	*F*	*p*	Partial η²
Within-subjects effects						
Time interval	1.58	1	1.575	6.94	0.009	0.037
Time interval × resin composite	11.74	4	2.936	12.95	< 0.001	0.223
Time interval × immersion solution	11.48	3	3.825	16.86	< 0.001	0.220
Time interval × resin composite × immersion solution	25.37	12	2.114	9.32	< 0.001	0.383
Residual	40.83	180	0.227	–	–	–
Between-subjects effects						
Resin composite	470.0	4	117.489	321.3	< 0.001	0.877
Immersion solution	293.3	3	97.769	267.3	< 0.001	0.817
Resin composite × immersion solution	427.5	12	35.627	97.4	< 0.001	0.867
Residual	65.8	180	0.366	–	–	–

Because Levene’s test indicated heterogeneity of variances for the ΔE_00_ intervals, Games–Howell post-hoc comparisons were used for pairwise comparisons among composite–solution combinations. Post-hoc grouping symbols for T0–T1 and T0–T2 are presented in Table 3.

### Whiteness Index (WID) results

**Figure 4:**
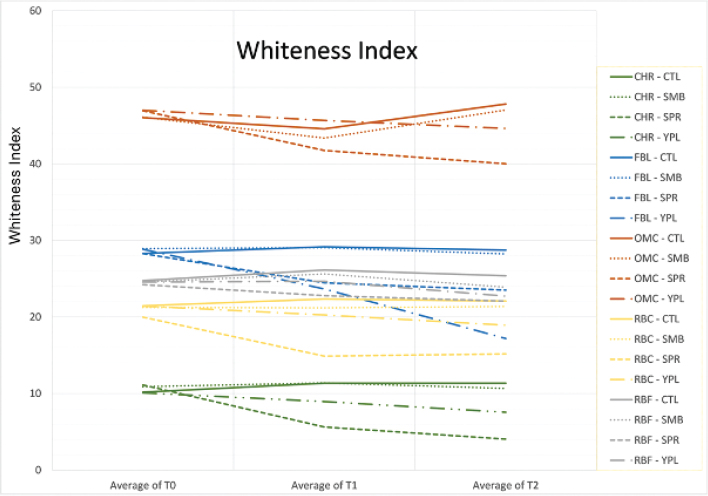
Whiteness index changes over time. Baseline (T0), after 14 days (T1), and after 28 days (T2). The resin composites tested were Charisma Smart (CHR), Filtek One Bulk Fill (FBL), Omnichroma (OMC), Ruby Comp Nano (RBC), and Ruby Flow (RBF). The immersion solutions were distilled water control (CTL), Sambucol Plus (SMB), Supradyn Effervescent Tablet (SPR), and Youplus Vitamin C Zinc Propolis effervescent tablet (YPL). The different line styles represent each resin composite-solution combination.

While ΔE_00_ served as the primary color change indicator, WID was calculated as a supplementary measure to assess esthetic shifts. The WID values shown in Figure 4 generally decreased over time, with variations depending on the resin composite and solution used.

The ΔWI_D_ results are presented in Table 5. Negative ΔWI_D_ values indicate a reduction in perceived whiteness compared with baseline. Welch’s one-way ANOVA showed significant differences among the composite–solution groups for both ΔWI_D_ T0–T1 and ΔWI_D_ T0–T2 intervals. The group effect was significant for ΔWI_D_ T0–T1, *F*(19, 66.1) = 18.4, *p* < 0.001, and for ΔWI_D_ T0–T2, *F*(19, 66.0) = 14.3, *p* < 0.001.

**Table 5:** Changes in Whiteness Index for Dentistry (ΔWID) values of resin composites according to solution and time.

Resin composite	Solution	*n*	ΔWI_D_ T0–T1 mean ± SD	95% CI	ΔWI_D_ T0–T2 mean ± SD	95% CI
CHR	**CTL**	10	1.17 ± 2.07^abc^	−0.31 to 2.65	1.18 ± 2.06^a^	−0.29 to 2.65
CHR	**SMB**	10	0.50 ± 2.16^abc^	−1.05 to 2.05	−0.23 ± 2.13^ab^	−1.75 to 1.29
CHR	**SPR**	10	−5.48 ± 4.50^bcde^	−8.70 to −2.26	−7.06 ± 3.43^cd^	−9.51 to −4.61
CHR	**YPL**	10	−1.13 ± 2.53^abcd^	−2.94 to 0.68	−2.53 ± 2.78^abc^	−4.52 to −0.54
FBL	**CTL**	10	0.86 ± 2.21^abc^	−0.72 to 2.44	0.45 ± 2.73^a^	−1.50 to 2.40
FBL	**SMB**	10	0.10 ± 0.99^abc^	−0.61 to 0.81	−0.71 ± 0.79^ab^	−1.28 to −0.14
FBL	**SPR**	10	−3.82 ± 1.36^bcde^	−4.79 to −2.85	−4.76 ± 1.99^bc^	−6.18 to −3.34
FBL	**YPL**	10	−5.18 ± 0.87^e^	−5.80 to −4.56	−11.70 ± 2.58^d^	−13.55 to −9.85
OMC	**CTL**	10	−1.48 ± 5.49^abcde^	−5.41 to 2.45	1.78 ± 2.76^a^	−0.19 to 3.75
OMC	**SMB**	10	−2.74 ± 7.10^abcde^	−7.82 to 2.34	0.95 ± 3.06^a^	−1.24 to 3.14
OMC	**SPR**	10	−5.16 ± 3.06^bcde^	−7.35 to −2.97	−6.92 ± 6.85^bcd^	−11.82 to −2.02
OMC	**YPL**	10	−1.32 ± 3.01^abcde^	−3.47 to 0.83	−2.38 ± 3.61^abc^	−4.96 to 0.20
RBC	**CTL**	10	0.89 ± 1.75^abc^	−0.36 to 2.14	0.64 ± 2.43^a^	−1.10 to 2.38
RBC	**SMB**	10	−0.04 ± 2.69^abc^	−1.96 to 1.88	0.15 ± 2.71^ab^	−1.79 to 2.09
RBC	**SPR**	10	−5.08 ± 1.84^de^	−6.40 to −3.76	−4.81 ± 2.94^bc^	−6.91 to −2.71
RBC	**YPL**	10	−1.17 ± 2.43^abc^	−2.91 to 0.57	−2.52 ± 2.20^abc^	−4.09 to −0.95
RBF	**CTL**	10	1.39 ± 1.93^a^	0.01 to 2.77	0.62 ± 1.44^a^	−0.41 to 1.65
RBF	**SMB**	10	1.08 ± 2.08^abc^	−0.41 to 2.57	−0.66 ± 2.00^ab^	−2.09 to 0.77
RBF	**SPR**	10	−1.44 ± 2.01^abc^	−2.88 to −0.004	−2.15 ± 2.18^abc^	−3.71 to −0.59
RBF	**YPL**	10	0.10 ± 1.07^abc^	−0.67 to 0.87	−1.86 ± 2.25^abc^	−3.47 to −0.25

SD: standard deviation; CI: confidence interval; CHR: Charisma Smart; FBL: Filtek One Bulk Fill; OMC: Omnichroma; RBC: Ruby Comp Nano; RBF: Ruby Flow; WID: Whiteness Index for Dentistry; CTL: distilled water control; SMB: Sambucol Plus; SPR: Supradyn Effervescent Tablet; YPL: Youplus Vitamin C, Zinc, Propolis effervescent tablet.

Values are presented as mean ± standard deviation and 95% confidence interval. Superscript lowercase letters indicate Games–Howell post-hoc groupings within each time interval. Values sharing at least one common letter within the same time interval are not significantly different from each other (*p* > 0.05). T0–T1 and T0–T2 post-hoc groupings were calculated separately.

Overall, the greatest reductions in WI_D_ were observed in the SPR- and YPL-exposed groups, whereas the control groups generally showed minimal changes or slight increases in WID. At T0–T1, the most pronounced decreases were mainly observed in the SPR groups and in the FBL–YPL group. At T0–T2, FBL–YPL showed the greatest reduction in whiteness, followed by the SPR-exposed groups, particularly CHR–SPR, OMC–SPR, RBC–SPR, and FBL–SPR.

Games–Howell post-hoc comparisons confirmed significant differences among several composite–solution combinations at both time intervals. In general, YPL produced the most marked WI_D_ reduction in FBL after 28 days, while SPR caused consistent reductions across multiple resin composites. The distilled water control groups remained comparatively stable throughout the observation period.

### Visual representation of color changes

Supplementary Figure S1 provides a visual representation of the color changes in the tested resin composites across the three evaluation periods (T0, T1, and T2). The figure is presented as a qualitative aid to facilitate interpretation of the instrumental color measurements.

### pH measurements

The initial pH values of the multivitamin solutions were measured. The SMB solution had an initial pH of 4.4, the SPR solution had a pH of 4.5, and the YPL solution had a pH of 3.9. These data indicate that all three multivitamin solutions were acidic, exhibiting distinct degrees of acidity.

## Discussion

As the use of effervescent vitamins becomes increasingly common among individuals [21], understanding their potential impact on dental restorations is crucial. Unlike well-known staining agents such as coffee or tea, the effects of these supplements on resin composites remain under-researched, despite their regular use in daily life [6, 22, 23].

The present study aimed to fill this gap by evaluating the effect of different multivitamin solutions on the color stability of various resin composites. Based on the results, the null hypothesis – that multivitamin effervescent tablets do not affect the color change of resin composites – was rejected. Statistically significant discoloration was observed in the FBL and OMC resin composite, especially following exposure to YPL and SPR solutions. The ΔE_00_ values for these groups exceeded the acceptability threshold of 1.8, suggesting that such changes would be noticeable in the oral environment.

The acidic nature of the effervescent solutions, particularly YPL (pH 3.9) and SPR (pH 4.5), may have contributed to the observed color changes. Low-pH environments have been associated with surface alterations in resin-based materials [24–26]; however, acidity should be interpreted as only one possible contributing factor in the present study, since titratable acidity and surface properties were not evaluated. In addition to pH, the colorants, additives, and specific composition of each effervescent formulation may also have influenced the discoloration pattern. In a previous study, Redoxon Triple Action (pH 3.0) was reported to significantly affect the color and surface roughness of dental resin composites over time [25].

Although the mechanisms responsible for discoloration were not directly investigated, previous studies suggest that acidic media and staining agents may affect resin composite matrices, potentially increasing staining susceptibility and altering optical properties. Such effects may be particularly relevant for materials such as Omnichroma, which rely on structural color and light-scattering mechanisms for shade matching. Therefore, the proposed explanations should be regarded as possible mechanisms rather than confirmed findings, as no physicochemical or surface analyses were performed in the present study.

Omnichroma is a single-shade resin composite restorative material designed to match Vita Classical shades, from A1 to D4 [27]. Its matrix, composed of lower molecular weight monomers such as urethane dimethacrylate (UDMA) and triethylene glycol dimethacrylate (TEGDMA), is prone to increased water sorption due to the hydrophilic nature of TEGDMA, potentially compromising color stability [28]. Studies have shown that Omnichroma exhibits lower color matching and more staining in cola and coffee compared to multi-shade resin composites. This increased staining may be related to its high-water absorption and the oxidation of unreacted double-bond methyl groups, which may lead to functional group changes that weaken light reflection, consistent with the hydrophilic properties of TEGDMA [29, 30].

Also, the unique filler compositions and resin matrices of tested resin composites may inherently possess higher susceptibility to staining. The high level of discoloration seen in FBL, for instance, could be linked to its bulk-fill nature, which is designed to allow polymerization up to five mm thickness. Bulk-fill resin composites exhibit distinct characteristics when compared with traditional resin-based composites [31]. Their matrices are typically cross-linked networks designed to accommodate polymerization-induced stress, while modified fillers optimize light transmission depth. Manufacturers meticulously match filler and matrix refractive indices to attain heightened translucency and conversion efficiency [32]. The susceptibility of bulk-fill resin composites to discoloration is correlated with the chemical composition and concentration of resin monomers. The resin matrix’s affinity for staining pigments is modulated by its degree of conversion and physical attributes, such as water sorption. This latter property can compromise resin longevity by inducing hydrolysis of silane coupling agents, and subsequent stain penetration [31].

Notably, the distilled water control group showed measurable color changes in some resin composites, particularly after 28 days. This finding suggests that water sorption alone can contribute to color alterations in certain resin composite formulations. The hydrophilic nature of some resin monomers, particularly TEGDMA, may facilitate water uptake leading to matrix swelling and optical property changes as stated above.

As shown in Supplementary Figure S1, the color blocks demonstrate the variations in color for each resin composite-solution combination over time, highlighting the extent of discoloration, particularly in resins exposed to YPL and SPR solutions, which show noticeable changes by T2. The displayed colors serve primarily as a visual aid to illustrate the relative magnitude and direction of color changes between different treatment groups rather than exact color reproduction.

The variation in response among different resin composites, such as the relatively better color stability observed in Charisma Smart (CHR), Ruby Comp Nano (RBC), and Ruby Flow (RBF), suggests that factors beyond mere exposure to acidic environments are at play. Differences in filler particle size, distribution, and the chemical nature of the monomers used in these resin composites likely influence their resistance to staining.

Several limitations should be considered when interpreting the findings of this study. First, although the immersion protocol was designed to simulate daily effervescent tablet consumption, the in vitro model did not reproduce important oral conditions such as saliva, acquired pellicle formation, toothbrushing, thermocycling, biofilm, mechanical wear, or dietary interactions. Therefore, the discoloration patterns observed under laboratory conditions should not be directly extrapolated to clinical situations.

Second, the specimens were polymerized against Mylar strips and were not subjected to finishing or polishing procedures. This approach allowed standardization of the specimen surfaces and reduced methodological variability; however, clinically placed resin composite restorations usually undergo finishing and polishing, which may affect surface roughness, gloss, and staining susceptibility.

Third, no complementary analyses, such as surface roughness, microhardness, scanning electron microscopy, water sorption, solubility, or degree of conversion measurements, were performed. Therefore, the mechanisms underlying the observed color changes cannot be directly confirmed and should be interpreted only as possible explanations based on previous evidence. In addition, pH was measured only once at baseline, and titratable acidity was not evaluated; therefore, the reported pH values should be regarded as descriptive indicators rather than comprehensive measures of erosive or staining potential.

Within these limitations, the findings suggest that frequent exposure to effervescent multivitamin solutions may influence the color stability of certain resin composites. Further in situ and clinical studies incorporating saliva, brushing, thermocycling, finishing/polishing protocols, repeated pH measurements, titratable acidity, and surface analyses are needed to confirm the clinical relevance of these results.

## Conclusions

Within the limitations of this in vitro study, exposure to multivitamin effervescent solutions resulted in material- and solution-dependent color changes in resin composites. Filtek One Bulk Fill and Omnichroma showed the greatest discoloration, particularly after exposure to Youplus and Supradyn solutions. Although some color changes were also observed in distilled water, the effervescent multivitamin solutions produced more pronounced alterations under the present laboratory conditions.

These findings suggest that effervescent multivitamin solutions may affect the color stability of certain resin composites. However, direct extrapolation to clinical conditions should be made cautiously, as the experimental model did not fully reproduce the oral environment, including saliva, pellicle formation, toothbrushing, thermocycling, biofilm, or dietary interactions.

## Supplementary Material



## Data sharing statement

The datasets are not publicly available but are available from the corresponding author upon reasonable request.
