# Supplementary material for: Effect of effervescent multivitamin solutions on the color stability of contemporary resin composites: an in vitro study
Source: Biomater Investig Dent. 2026 Aug 4;13:46591. doi: 10.2340/biid.v13.46591 (PMC13449964; doi:10.2340/biid.v13.46591)

Supplementary material has been published as submitted. It has not been copyedited or typeset by Biomaterial Investigations in Dentistry.

Supplementary Figure 1

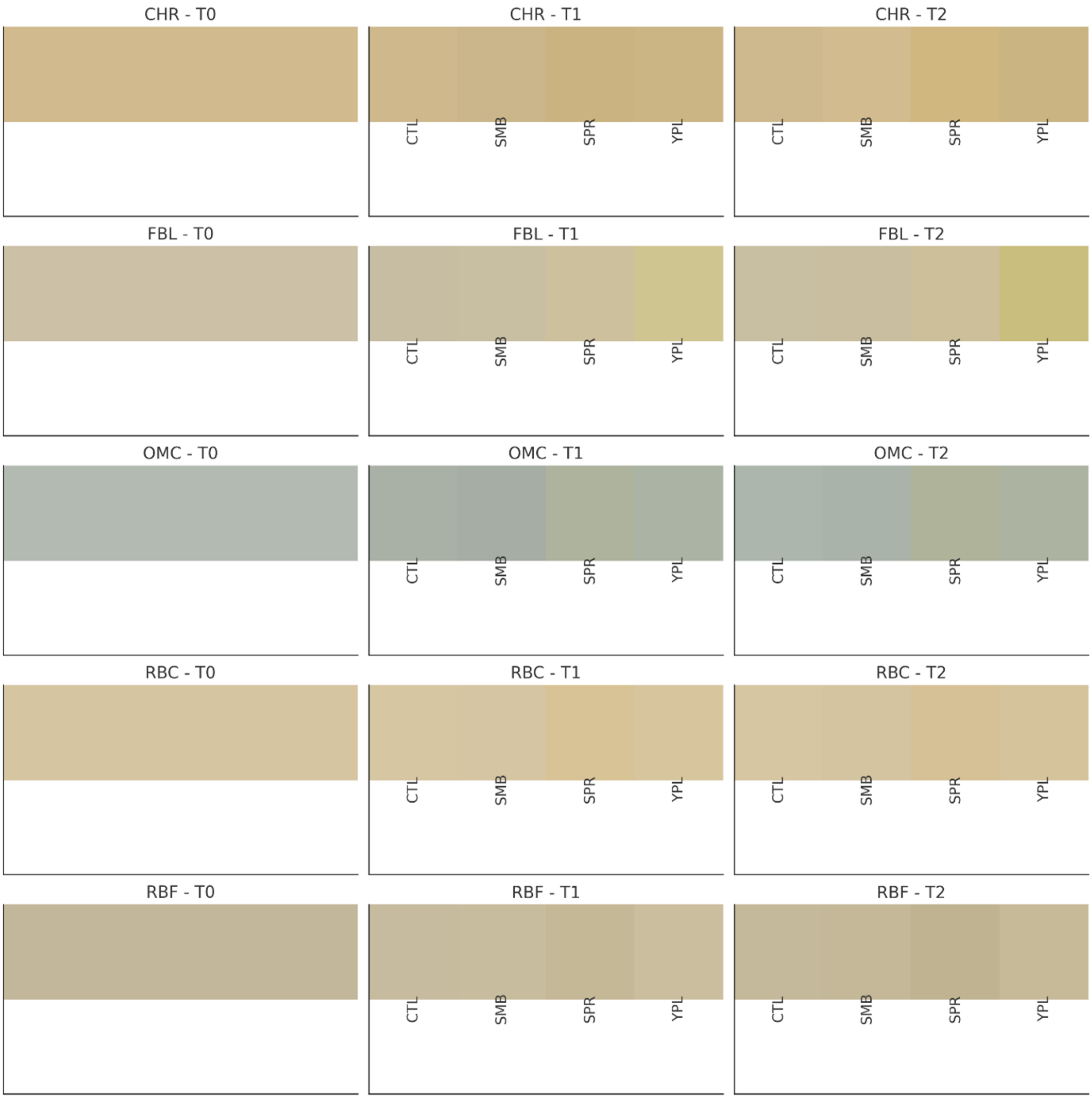

Supplement: Supplementary file 1 [file BIiD-13-46591-s1.pdf]
